# Severe and Enduring Eating Disorders: A Qualitative Study of Patients' Experiences and Perceived Treatment Needs

**DOI:** 10.1002/erv.70132

**Published:** 2026-06-01

**Authors:** Emilia Kotilahti, Anu Ruusunen, Katarina Lindstedt, Rasmus Isomaa

**Affiliations:** ^1^ Institute of Public Health and Clinical Nutrition, University of Eastern Finland Kuopio Finland; ^2^ Mental Health and Wellbeing Kuopio University Hospital, Wellbeing Services County of North Savo Kuopio Finland; ^3^ Deakin Institute for Mental and Physical Health and Clinical Translation (Deakin IMPACT) Food & Mood Centre, School of Medicine, Deakin University Geelong Australia; ^4^ University Health Care Research Center, Faculty of Medicine and Health Örebro University Örebro Sweden; ^5^ Fredrika Clinic, The Wellbeing Services County of Ostrobothnia Jakobstad Finland

**Keywords:** eating disorder, lived experiences, patient perspective, severe and enduring, thematic analysis, treatment

## Abstract

**Objective:**

Approximately 25% of individuals with eating disorders develop severe and enduring eating disorders (SEEDs); yet effective treatments remain limited. This study explored lived experiences of SEEDs, focussing on past treatments, as well as current needs and hopes.

**Method:**

Twenty participants with eating disorders of ≥ 7 years and prior treatment engagement were interviewed about treatment experiences and needs. Transcribed interviews were analysed using phenomenologically informed thematic analysis.

**Results:**

Four main themes emerged: (1) treating the weight, the illness or the person; (2) the attitude of the professionals; (3) the need for specialised treatment; and (4) losing control. The first theme comprised sub‐themes: (a) too much focus on weight, (b) one size doesn't fit all, and (c) finding and connecting with the person. Participants found individual tailored treatments, multidisciplinary teams, and specialised ED treatments beneficial. Many felt that their unique situation and needs were overshadowed by a predominant emphasis on weight or inflexible treatment protocols.

**Conclusions:**

The findings support evidence suggesting that treatment should shift from a primary focus in body weight towards more individualised approaches that promote sustainable care and strengthen therapeutic alliances. Future research should examine ways for balancing necessary restrictions with flexibility in inpatient and involuntary care.

## Highlights

- Findings highlight the need to shift treatment paradigms from weight‐centric, uniform models towards individualised approaches that integrate psychiatric comorbidities and life complexities to support continuity of care and long‐term outcomes.
- Limited professional knowledge and resources hindered supportive care, and clinicians' attitudes and patient‐provider alignment critically shaped patients' experiences and recovery.
- Inpatient and involuntary care were perceived predominantly negatively mainly due to loss of control, though its nutritional benefits were acknowledged later, pointing to the value of more balanced and flexible strategies.

## 1 Introduction

Eating disorders (EDs) are serious mental illnesses; according to recent estimates, about 25% of all cases persist and become treatment refractory (Solmi et al. 2024). Recently, treatment options for severe and enduring eating disorders (SEEDs) have been widely discussed although SEED definition and labelling are still somewhat unclear (Broomfield et al. 2017). While SEEDs were initially conceptualised primarily in relation to anorexia nervosa—and most research has focused on individuals with this diagnosis (Broomfield et al. 2017; Strober 2004; Yager 2020)—emerging evidence indicates that bulimia nervosa can also follow chronic and severe trajectories (Robinson et al. 2024). People with SEEDs might benefit from, instead of traditional treatment goals such as intensively improving nutritional status, a modified approach (Strober 2004; Wonderlich et al. 2012). As EDs lengthen, they increasingly shape patients' lives and identities, fostering reluctance towards change (Fox and Diab 2015). Thus, to patients, recovery might seem at least as distressing as the suffering caused by the disorder. Too strict treatment goals might promote resistance and lead to dire consequences, even suicide (A. R. Smith et al. 2018). Therefore, treatment of patients with SEEDs could be centred upon vocational and social issues as well as enhancing patients' quality of life (Wonderlich et al. 2012). To date, the only randomised controlled trial involving individuals with SEEDs demonstrated that shifting the treatment focus towards quality‐of‐life improved treatment engagement and motivation, while also reducing eating disorder pathology (Touyz et al. 2013).

Despite these developments, existing studies on SEEDs have primarily focused on traditional outcomes such as weight restoration and symptom reduction (Yager 2020). At the same time, the controversial concept of terminal anorexia nervosa—referring to a condition understood to lead to death—has generated ethical debate regarding end‐of‐life care, appropriate treatment models (e.g., harm reduction, recovery‐oriented approaches, and palliative care), and their implications for perceptions of hope and hopelessness (Kiely et al. 2025). Importantly, harm‐reduction and recovery‐oriented frameworks are not mutually exclusive and may be implemented concurrently (Gilmartin et al. 2025). Within this context, various treatment approaches—ranging from inpatient and outpatient care to pharmacological and brain stimulation therapies—have been explored, yet optimal care remain uncertain (Kotilahti et al. 2020). Clinicians emphasise the importance of listening to patients and collaboratively defining treatment goals (Tierney and Fox 2009; Wonderlich et al. 2012). However, patients and healthcare professionals have been shown to differ in their conceptualisations of recovery (Ferreira et al. 2025).

Few studies have explored these factors from patients' perspectives. Robinson et al. (2015) interviewed eight individuals with EDs of over 20 years' duration, including three outpatients, 2 day patients, and three inpatients, with a focus on symptoms, behaviours, and treatment experiences. The study was preliminary, as data saturation was not reached, but indicated that patients with SEEDs seek support, attachment and even friendship within treatment. The importance of personal connection and collaborative relationships in fostering treatment engagement has also been supported by other studies involving SEED patients currently undergoing inpatient care (Cummings et al. 2023; Fox and Diab 2015).

Although the treatment of SEEDs remains debated, empirical research is scarce. Given the limited evidence to guide treatment, research into patients' experiences and perceptions of their illness is essential (Conti et al. 2016). In particular, our phenomenologically informed thematic analysis, conducted with participants both with and without current treatment involvement, enables the generation of rich and nuanced accounts that foreground lived experience, emotional responses, and personal meaning‐making. Such insights complement existing research by illuminating dimensions of experience that quantitative methods are not designed to capture (Braun and Clarke 2006; Earle 2010).

## 2 Method

### 2.1 Participants

Participants were considered eligible if they had received treatment for an ED and at least 7 years had elapsed since initial treatment. We included only adult participants with typical or atypical anorexia nervosa (AN) or bulimia nervosa (BN) as their primary diagnosis who self‐identified themselves as having or having had a SEED. Individuals with a binge eating disorder (BED) or eating problems secondary to other disorders were excluded.

The final sample comprised 20 participants with a mean age of 32 years (range 22–58) and a mean age of ED onset of 12. All, but one non‐binary participant, identified as women. Diagnostic and treatment information was self‐reported, as medical records were not accessible. Treatment duration ranged from less than a year to 12 years, with a mean of 8 years.

Participants considered themselves to be at varying stages of treatment and recovery. Three participants (15%) reported full recovery and three reported absence of current symptoms, whereas half (*n* = 10) indicated ongoing symptoms of varying severity. The majority (*n* = 13) described ED‐related thoughts. Further details about participant demographics, their treatments, and comorbidities are provided in Tables 1 and 2.

**TABLE 1:** Participant demographics.

Characteristics	*n* (proportion)
Current occupation^a^	
Employed	10 (50%)
Student	4 (20%)
Retired	4 (20%)
Parental leave	1 (5%)
Rehabilitation	1 (5%)
Frequence of work/studies^a^	
Working and/or studying 50% or more	16 (80%)
Working and/or studying less than 50%	3 (15%)
Not working or studying	1 (5%)
Education	
University or university of applied sciences	15 (75%)
General or vocational upper secondary school	4 (20%)
Comprehensive school	1 (5%)
Main source of income	
Salary from employment or entrepreneurship	10 (50%)
Pension	6 (30%)
Study grant	2 (10%)
Parental allowance	1 (5%)
Social assistance	1 (5%)
Marital status	
Single	8 (40%)
Living with a partner	6 (30%)
In a relationship	4 (20%)
Married	1 (5%)
Divorced	1 (5%)
Living situation^a^	
Living alone	8 (40%)
Living with a partner	7 (35%)
Living with a child or children	3 (15%)
Living with a parent or parents	1 (5%)
Living with another adult and my own child or children	1 (5%)
Children	
No	14 (70%)
Yes	6 (30%)
Ethnicity	
White	20 (100%)
Eating disorder diagnosis	
Anorexia nervosa	15 (75%)
Bulimia nervosa	4 (20%)
Atypical anorexia nervosa	1 (5%)
Comorbid psychiatric and neuropsychiatric diagnosis	
Depression	14 (70%)
Anxiety	7 (35%)
Any personality disorder	5 (25%)
Posttraumatic stress disorder	3 (15%)
Panic disorder	2 (10%)
Autism	1 (5%)
Other	3 (15%)
None	3 (15%)

^a^ Over the past 12 months.

**TABLE 2:** Participants' treatment history.

	Yes, *n* (proportion)
Prior treatments	
Nutritional therapy	19 (95%)
Psychotherapy	17 (85%)
Physical therapy	16 (80%)
Drug therapy	15 (75%)
Visits to primary healthcare^a^	15 (75%)
Health monitoring in primary healthcare^a^	14 (70%)
Occupational therapy	9 (45%)
Brain stimulation therapy	2 (10%)
Other^b^	4 (20%)
Prior treatment regimens	
Outpatient treatment	19 (95%)
Inpatient treatment	16 (80%)
Day‐hospital treatment	14 (70%)
Prior nutritional rehabilitation	
Nutritional therapy	19 (95%)
A meal plan	17 (85%)
Oral nutritional supplements	13 (65%)
Enteral tube feeding	7 (35%)
Parenteral nutrition	7 (35%)
Involuntary treatment	12 (60%)
Breaks in treatment	15 (75%)

^a^ School, student health care or local health station.

^b^ Group therapy, online therapy, peer counselling, dance/movement therapy.

### 2.2 Procedure

The study was approved by the Ethics Committee of the Hospital District of Northern Savo (2191/2022).

Participants were recruited via the social media channels of The Eating Disorder Association of Finland (Syömishäiriöliitto, Syli ry), a national interest group for individuals with EDs and their families and friends. Recruitment was based on illness duration, which is the most commonly cited criterion for enduring EDs (Broomfield et al. 2017), thereby ensuring comparability with previous studies. Study information was first posted on Facebook and Instagram, including the contact details for the first author. Additional information was then provided to the 33 candidates who expressed interest in participation. Prior to the interviews, eligibility was assessed through a brief telephone screening. Twenty‐two participants met the inclusion criteria and provided written informed consent for participation and recording. Two participants later withdrew from the study. No monetary or other compensation was provided for participation.

Participants completed a web‐based questionnaire on background, health, and treatment history prior to a single semi‐structured interview. The questionnaire and interview guide were developed collaboratively by the research team, integrating expertise in psychology, psychiatry, and nutrition (see Supporting Information S1 and S2). Questionnaire responses were reviewed prior to each interview to allow for a more informed discussion. The first author (E.K.) conducted all the interviews, including a pilot session used to refine procedures, via Microsoft Teams to offer flexible, remote participation. The interviews explored participants' current situation, somatic health, treatment experiences, and perceived needs, and lasted 23–112 min. E.K. then transcribed the interview recording verbatim and replaced identifiable information with pseudonyms. The participants were encouraged to contact the researcher after the interview; two provided additional information via email. Atlas.ti software was used to support data management and coding.

### 2.3 A Qualitative Analysis

A thematic approach with phenomenological underpinnings was used to analyse the data. Phenomenology seeks to understand human experiences on their own terms, emphasising lived experiences and the interpretation of feelings as they arise and are subsequently reflected upon (Earle 2010; N. Smith et al. 2009). The exploratory aim and inductive, data‐driven thematic analysis (Braun and Clarke 2006) indicated the need for a sample sufficient to capture variation and support robust theme development. Although participants shared lived experience of SEEDs, anticipated heterogeneity in treatment trajectories and needs suggested moderate sample specificity. The use of in‐depth interviews enhanced data richness, and the final sample was considered to provide adequate informational depth for the purposes of the present analysis, consistent with the concept of information power described by Malterud et al. (2016).

To reduce bias and validate analysis, two independent authors (E.K. and R.I.) repeatedly read and familiarised themselves with all 20 transcripts and coded the data. Other authors (K.L. and A.R.) were provided with summarising texts of the interviews. The interviews were conducted and analysed in Finnish by the first and last author, while analytic discussions involving all authors were held in English. The first author translated the quotations and summary texts, and the last author verified their accuracy. Selected translations were reviewed in meetings with the full research team. We adopted an inductive, data‐driven approach, allowing themes to emerge from participants' accounts rather than from a predefined coding scheme or researcher hypothesis. Themes were analysed at the semantic level, focussing on explicit meaning without inferring latent content. The analysis was conducted within an essentialist paradigm, which assumes a direct and unidirectional relationship between experience and language.

Recurrent themes were identified following the six steps in thematic analysis adapted from the guidelines of Braun and Clarke (2006). The analytic process involved familiarising with the data (step 1), generating initial codes (step 2), and searching for themes within those codes (step 3). The tentative codes and themes were refined through repeated discussions and feedback (step 4), defined and named (step 5), involving three of the authors (E.K., R.I., and K.L.) to produce the final report (step 6). The iterative process involving revisiting and adjusting the initial themes based on collaborative input, aimed at more robust and well‐supported analysis. Direct quotations from transcripts were included to ensure credibility and convey participants' voices. To enhance transparency, E.K. maintained a thorough audit trail documenting analytic procedures and decision‐making, as recommended by Wolf (2003). Participant characteristics were reported to facilitate the understanding of the findings’ fittingness and transferability.

### 2.4 Reflexivity

E.K., who conducted the interviews, is a young, Caucasian woman at an early stage of her career. Her knowledge of EDs is limited to academic training and research, with minimal clinical and no personal experience in the field. This lack of lived experience may have limited participants' willingness to share certain perspectives as they might with a peer. Conversely, her relative inexperience may have enhanced her perceived approachability, potentially encouraging participants to discuss sensitive topics—such as challenges in treatment—that they might have been reluctant to disclose to a clinician involved in their care. However, limited experiential familiarity may also have resulted in some subtle cues being overlooked during interviews. To balance individual positionality, the research group includes persons with considerable academic and clinical experience with EDs (R.I., K.L., and A.R.). The authors' diverse backgrounds enhanced perspective and mitigate individual bias, while researchers' positionality was addressed through collaborative design and the maintenance of a research journal.

## 3 Results

The analysis identified four main themes and three sub‐themes, as illustrated in Figure 1. The figure presents four main themes on the left and the three sub‐themes branching from Theme 1.

**FIGURE 1:**
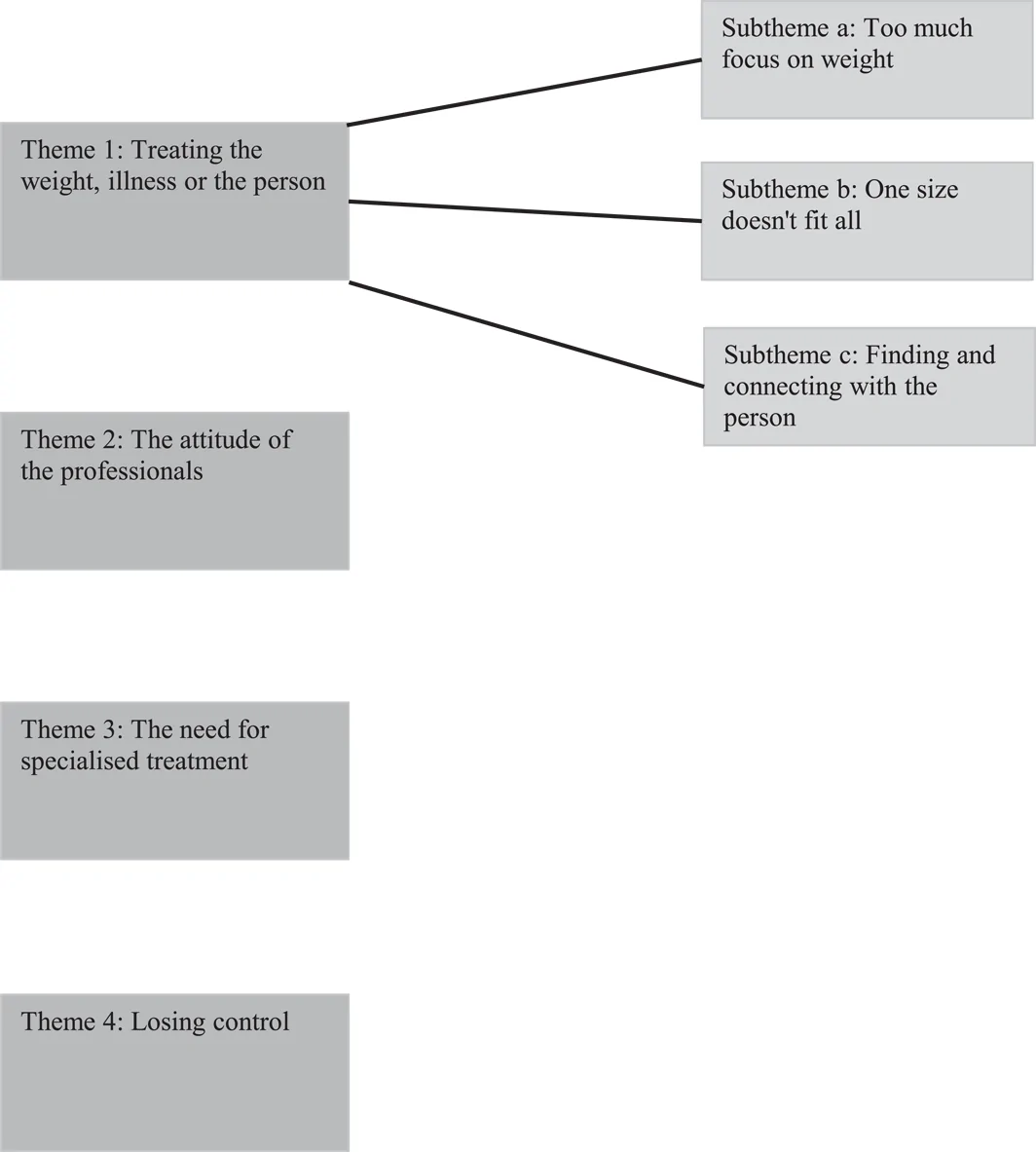
Main themes and sub‐themes.

### 3.1 Theme 1: Treating the Weight, the Illness or the Person

The participants identified three main areas of focus in their ED treatments: body weight, symptomatic manifestations, and the individual. However, treatment often prioritised weight and diagnostic criteria, with limited attention to individual needs and circumstances.

- Too much focus on weight

The participants perceived the excessive focus on weight as a highly negative aspect of their treatment experience. Some reported being denied access to treatment because their weight did not meet admission criteria, which they associated with worsening ED behaviour. Extremely underweight participants, in turn, acknowledged their privilege in accessing treatment as opposed to those with a BMI closer to the normal range.

Well, of course the never‐ending problem with eating disorders that they are perceived as disorders of the weight which they are not. So, at a younger age it was a big problem since my weight hadn’t dropped enough so the situation was considered to be not that bad. And that’s why I didn’t get help. And that was the problem also in the hospital as to why it wasn’t paid attention to, because I hadn’t lost enough weight.

Ida, 27 years old

Several participants reported that treatment was often discontinued once they reached a normal weight, leaving them feeling vulnerable and unsupported. Some expressed concern about the lack of treatment sustainability and the fear that care would end due to weight gain or low motivation. Constant weight monitoring was described as distressing and as limiting engagement with other aspects of treatment. The strong focus on weight was also perceived to obscure the recognition of EDs as mental illnesses, with some participants suggesting this contributed to relapse later in life.

And then like, so, the thing was the weighing. So, like the weighing preceded every appointment even though maybe some kind of conversation would have been more important after all. So, the weighing was like so distressing.

Maarit, 49 years old

Several participants pointed out that treatment and recovery should place less emphasis on body weight, noting that weight does not directly reflect symptom severity or its impact on daily life. They highlighted that while weight can change rapidly, genuine recovery is a more complex and prolonged process. Many suggested that weighing should be limited to somatic monitoring and play only a secondary role. One participant acknowledged that treatment had become less weight‐focused but stressed that further progress is needed.

b. One size doesn't fit all

The participants perceived the lack of individuality in treatment negatively. Their critique highlighted a deficiency in tailoring treatments to their specific needs and circumstances. Some reported that their diagnosis determined treatment eligibility, while strict policies prevented access to care, they considered necessary.

Because I feel like there in the eating disorder unit it was like so that according to your diagnosis you were put to a specific like framework. And everyone is like treated the same way if you have a certain diagnosis. Although everyone has a completely different situation after all.

Anna, 28 years old

Participants reported that treatments often prioritised diagnostic criteria over individual needs. Rigid adherence to diagnostic thresholds may therefore undermine engagement and recovery, underscoring the need for more flexible, person‐centred approaches that recognise psychological and behavioural recovery alongside physical indicators.

Experiences of peer support were mixed. For some, group participation was highly valuable, offering encouragement not only from others with EDs but also from those with different psychiatric conditions. For others, however, peer interactions were triggering, fostering unhealthy comparison and competition.

Regarding psychiatric comorbidity, some participants described receiving integrated care where all diagnoses were addressed, while others felt that their ED was overlooked in favour of other conditions. A few participants reported feeling ‘bounced’ between services due to multiple diagnoses and the lack of uniform treatment.

c. Finding and connecting with the person

Treatments that focused on overall well‐being, personal values and interests were described as particularly meaningful, supporting symptom reduction and, in some cases, recovery. Participants highlighted the importance of addressing underlying causes in therapy, noting that non‐ED specific psychotherapy was often effective in working with cognitions and vulnerabilities, though two participants reported negative experiences.

Healthcare professionals' genuineness, willingness to help, and the quality of therapeutic relationships were highly valued; encounters with individual professionals were sometimes described as crucial for recovery.

Well, when I was younger like out of everyone I saw, I can say that it was mainly thanks to this psychiatric nurse. And a big part of it was that we like as persons clicked so well, and then we talked a lot. And at that time theatre was important for me, and she was also a theatre‐person, and it was like an asset, you know. Sometimes we talked about that and those dreams, and then it was like, we focused on going forward.

Minna, 30 years old

Motivation was repeatedly underlined as essential: while treatment could provide insight, support, and tools, participants emphasised that recovery ultimately depended on a strong personal desire to change. Many expressed hope that future treatments would place greater focus on the person behind the illness.

And sort of all that kind of treatment, where the individuality has been involved, so, I feel, that it has taken me forward.

Satu, 36 years old

Well yeah, I was the main initiator. To become my own person, so to say. And to go through all the childhood wounds and pain since I had the understanding. The one about all the problems we have. So yes, the foundation is there in the childhood.

Leena, 58 years old

And sort of like through that sort of a treatment I’ve learnt to understand like myself and my symptoms and all that like a lot better. So that has at least been something that I’ve experienced to be like important.

Satu, 36 years old

As illustrated in the quotations above, participants described treatment as pivotal in fostering increased self‐awareness, including insights into etiological processes and present circumstances, which subsequently supported the development of recovery‐related self‐efficacy.

### 3.2 Theme 2: The Attitude of the Professionals

Participants described professionals and treatment units as having a major influence on their care, with both positive and negative experiences often occurring within the same unit. Central to both experiences were whether participants felt ‘heard’ and ‘taken seriously’, with compassion regarded as an important professional quality.

But the kind that if you like get the feeling that you are met as a person and not just as a difficult patient with an eating disorder. So, that has maybe been it, where I haven’t had such feelings of resistance towards treatment.

Kuura, 25 years old

Healthcare professionals were predominantly perceived as supportive and kind. Negative experiences, however, included inappropriate commenting about bodies, food and prognosis, as well as excessive control within the unit or from specific team members. While strictness was sometimes viewed positively, some participants reported anger, frustration, or even ridicule from staff, which could feel threatening.

And in this situation where my weight was normal and had stayed so for a couple of weigh‐ins, the treatment was stopped. Sure, there was a summer break, and I once went to the eating disorder unit to talk about starting to binge and purge again. And the nurse just said, she was a summer temp, that eat gum when you feel like binging.

Karo, 26 years old

Expertise in EDs was seen to vary across units and among professionals. Experience was valued, but participants stressed that a basic understanding and empathetic attitude could also be sufficient for good care.

I guess basically the fact that those people that I’ve worked with have had some kind of an understanding about the disorder. You don’t have to have decades of experience in treating eating disorders to be able to create sort of a good relationship and meet the patient or the client genuinely and listen to them. But like a certain understanding of the working mechanisms behind the disorder has indeed been useful.

Stella, 31 years old

Conversely, lack of expertise and limited resources were described as major barriers. Participants felt that inexperience impeded connection and compassion, while resource shortages resulted in long waiting times, fragmented care, and treatment discontinuities. In some cases, ED symptoms escalated during delays, and one participant reported treatment being discontinued because another patient was deemed more severely ill. Across all levels of care, inadequate expertise and resources were perceived as negatively affecting treatment quality and delaying recognition of EDs.

Because when you’re really ill and in a poor physical state and then you get the experience, that nobody understands me. And these people, that are supposed to help me, don’t understand. So, I feel like that can make the situation so much worse.

Jenna, 22 years old

Well, at least that the person with a disorder is seen as a person and not just like as the eating disorder. So, that is at least that sort of a thing that, I think, is easily forgotten in treatment.

Emi, 27 years old

So, that’s why it would be great if people were educated more. Since eating disorders are really common and really dangerous illnesses. So that is maybe sort of an important thing.

Jenna, 22 years old

The quotations suggest that healthcare professionals' ability to see patients as individuals and to understand their lived experiences was viewed as potentially beneficial for their well‐being, highlighting the need for continuous development of professional expertise. Alongside clinical knowledge, empathy and authentic engagement emerged as central to the quality of treatment outcomes and the therapeutic alliance.

### 3.3 Theme 3: The Need for Specialised Treatment

Participants generally found individualised approaches, multidisciplinary teams, and specialised ED treatment beneficial. They valued pragmatic methods that provided concrete tools for managing difficult situations, and many regarded the direct focus on eating behaviour as a key advantage of inpatient care.

Well, definitely that I’ve in recent years received so individualised treatment, so I’ve had my own meal plans, and the pace has been much slower in treatment.

Hilda, 33 years old

Some, however, criticised sessions with psychiatric nurses, social workers, or psychologists as unfocused and ineffective. Consistent and coordinated care over time was described as especially valuable, though a few participants attributed success mainly to their own receptiveness and timing.

Looking forward, most participants emphasised the need for more individualised and flexible treatment, taking into account each patient's unique background, circumstances, strengths, and sources of motivation. They called for flexibility in areas such as meal plans, treatment duration, and methods, as well as recognition of unhelpful differences in treating adult versus young patients.

So, sort of, I feel, that it would be really important to understand that you can’t treat teenagers exactly the same way as adults like. In a way adults can’t be like, since they have life behind them and they maybe know differently, what they want and all that, although, they have fallen ill.

Satu, 36 years old

So that it would be like more patient‐oriented. That they would be asked what kind of things they themselves might benefit from. And not in the way that we say: now we have this and this to offer, choose from these. That all treatment would kind of be available to everyone. Well, I understand that resources are resources and that there may not necessarily be more of them.

Anna, 28 years old

And then I’d hope just that there would be more treatment options for the ones with an enduring illness and sort of like although you wouldn’t be able to maintain your nutritional status or maintain weight, so you’d still get treatment. Or if you are like kind of labelled as chronic, then you’d still get some kind of support or treatment.

Hilda, 33 years old

The quotations above illustrate that strict treatment policies were widely opposed, and participants hope for expanded treatment options for those with enduring illness, regardless of their clinical history or nutritional status.

### 3.4 Theme 4: Losing Control

Participants described unpleasant experiences with involuntary treatment. Sixty percent of the participants reported having been ordered to treatment, while a few were uncertain whether their care had been voluntary. Involuntary treatment was often recalled as traumatic because of its unexpectedness, participants' young age or heightened sensitivity. Care was frequently provided in units lacking ED expertise, which was perceived as triggering and fostering resistance. Nonetheless, some participants acknowledged the necessity of involuntary treatment and even expressed gratitude, especially when it improved their nutritional status.

But despite that, I am anyway grateful for that my physical state was like improved during the treatment, although it didn’t take away the illness one bit. So, I am happy or like grateful for that, although I continue to think that both of those experiences were awful. And I sort of hope that they hadn’t happened.

Sara, 37 years old

Ambivalence was also reported regarding voluntary inpatient care. While seen as beneficial in some respects, extreme control was described as traumatic or exacerbating underlying trauma. Rules were sometimes perceived as arbitrary, constant suspicion as unjustified, and inpatient care during adolescence as disruptive to the process of independence. One participant felt that her entire identity had been lost in treatment.

Well, maybe what I have experienced, especially the treatment in the [Treatment Facility x] although there was a lot of expertise and such but there I was taken—so I feel—my whole identity was sort of taken from me. When trying to get rid of the eating disorder, remove all the ill routines and such. So, I feel that everything else was also taken. When sort of everything was forbidden, you couldn’t do anything. You can’t stand, you can only sit and eat. So, sort of that has stuck with me. I have even thought that it has been sort of traumatic for me that whole treatment. Because I have a lot of things running in my head still even though the treatment ended multiple years ago but still things come to mind daily.

Satu, 36 years old

The sense of control emerged as problematic: prior to treatment, control was largely held by the eating disorder itself, whereas during treatment, this control was transferred to healthcare professionals. Ideally, patients are expected to reclaim personal agency upon completing treatment. However, for some individuals, this transition was unsuccessful; instead of regaining control, the eating disorder symptoms often intensified shortly after discharge from involuntary or inpatient care.

And then now since my latest treatment period after which I was probably pretty close to my biological weight since the treatment period was like almost a year long after all. So, after that I just went downhill, and my weight dropped then after the treatment period actually to the lowest point that it had ever been, and it was for a while like so low that I could’ve been diagnosed with anorexia.

Satu, 36 years old

As the quotation above suggests, the perceived benefits of inpatient care were often temporary, with participants describing the eating disorder as quickly regaining an equal or stronger influence. These accounts of lost control point to the importance of carefully balancing clinical supervision with patient autonomy in promoting long‐term recovery.

## 4 Discussion

This study focused on the treatment experiences of individuals with lived experience of a SEED. Although the participants differed, for example, in the duration and initiation of their past treatments as well as in their current stage of recovery, their narratives revealed many shared experiences regarding past treatment and common hopes for future. These insights are essential for informing current treatment practices and for guiding professionals working in the field.

Perceptions of the disproportionate emphasis on physical symptoms, particularly body weight, align with results from previous ED research, especially those conducted in inpatient treatment settings (Colton and Pistrang 2004; Marzola et al. 2021; Offord et al. 2006; Rance et al. 2017). Rance et al. (2017), participants in this study reported that weight focus negatively affected treatment access, structure, and relationships. A key finding, also noted by Stockford et al. (2018), was that some participants with SEEDs perceived clinical emphasis on body weight as an implicit indication that their weight was not sufficiently low to be deserving of treatment. This perception, in turn, functioned as a reinforcing factor for further weight loss. Additionally, the participants reflected on the previously identified potential long‐term adverse effects associated with weight‐centric treatment approaches (Colton and Pistrang 2004). While starvation and malnutrition undoubtedly impair cognitive functioning (Fonville et al. 2014), the present findings support recent recommendations suggesting that, once medical stability is achieved, weight should not be the sole treatment target or outcome indicator in ED care (Phillipou et al. 2025).

Notwithstanding, the focus on body weight is an inherent feature of EDs and should be recognised as such. Therefore, the emphasis on weight cannot be attributed merely to treatment practices or healthcare professionals but patients themselves may contribute to shaping a treatment environment in which body weight becomes central. Nevertheless, a more comprehensive approach to identifying, understanding, and treating EDs is warranted (Phillipou et al. 2025). One possible strategy, also implicitly derived from our results, is to shift focus from body weight to eating behaviour—an aspect that patients can directly influence.

The participants in this study expressed varying perspectives on peer support: some experienced it as beneficial, while others perceived it as fostering unhealthy comparing and competition. This highlights the need for carefully structured group interventions with appropriate guidance and monitoring, as the social environment can function as both a therapeutic agent and a potential risk factor in recovery (Colton and Pistrang 2004; LaMarre et al. 2024). Equal recognition of all ED diagnoses is needed and establishing therapeutic connections that transcend diagnostic labels remain critical for effective care. As with previous research on patients with SEEDs (Cummings et al. 2023; Fox and Diab 2015; Robinson et al. 2015) as well as those with shorter duration of an ED (Lindstedt et al. 2015; Rance et al. 2017), participants wanted to be seen on their own and to form a genuine relationship with their therapist. Such relationships supported reflection on the past, self‐understanding, and future planning. Overall, the findings highlight the need to move beyond one‐size‐fits‐all models towards more nuanced and individualised approaches that account for possible psychiatric comorbidities and the complexities of each person's life.

As reported in previous research on SEEDs (Broomfield et al. 2021; Stockford et al. 2018), the attitudes of healthcare professionals and their level of expertise in EDs were perceived to significantly influence participants' treatment experiences and the overall quality of care. Similar patterns have been observed in adolescents with shorter illness duration, where therapists' attitudes form the foundation of the therapeutic relationship (Lindstedt et al. 2015). A perceived lack of professional knowledge and limited resources were in this study seen as barriers to adequate support and compassion, which in turn reinforced participants' pre‐existing feelings of being misunderstood and isolated. The experiences resonate with earlier findings indicating that social isolation and feelings of worthlessness are common among individuals living with SEEDs (Cummings et al. 2023; Stockford et al. 2018). Some participants reported overtly inappropriate comments or treatment, which they found particularly harmful. In contrast, therapeutic relationships in which they felt genuinely seen and heard were highly valued. Beyond expertise, factors such as interpersonal rapport and patient‐provider alignment are considered crucial to therapeutic alliance (Cummings et al. 2023). Thus, while professional expertise is valuable, it alone does not guarantee effective or compassionate care.

One of the most notable findings of this study was the overall negative perception of both involuntary and voluntary inpatient treatment. Over half of the participants had undergone formal compulsory treatment, and additional individuals reported experiences of coercion or threats. Negative emotions were linked to feelings of lost control and excessive regulation resonating with the adverse experiences documented by Mac Donald et al. (2023). Yet, as in that study, participants' attitudes often evolved over time. Recovery status (Mac Donald et al. 2023) and motivation (Colton and Pistrang 2004) have been shown to shape treatment acceptance, though ambivalence may persist even when individuals are assessed ready for change. Many participants retrospectively acknowledged the necessity of inpatient or involuntary care, particularly its role on improving nutritional status. This ambivalence is corroborated by a systematic review and meta‐synthesis by Rankin et al. (2023), that reported that patients recognise the value of inpatient care, while criticising its restrictive environment and narrow focus on physical health.

Consistent with Offord et al. (2006), participants also expressed a need for greater support following discharge, despite limited receptiveness at the time. These findings underscore the importance of sustained therapeutic relationships and structured discharge planning. Rather than ending treatment abruptly, ongoing contact—such as monthly follow‐up sessions—may better support long‐term recovery and can be delivered cost‐effectively.

## 5 Strengths and Limitations

This study has several strengths, some of which are its systematic qualitative method and the focus on lived experiences by which a deeper understanding of the participants' perspectives was gained. The participants were recruited via The Eating Disorder Association of Finland which enabled the inclusion of individuals both in treatment and outside the treatment system. Additionally, we included participants with both anorexia and bulimia nervosa, as well as participants in the various stages of illness and recovery to enhance the versatility of the findings. The inclusion of participants at different stages of illness and recovery provided a range of perspectives on long‐term illness trajectories and treatment experiences; however, this heterogeneity may limit the specificity of conclusions regarding individuals with current SEED. The identification of SEED relied on participants' self‐reported experiences rather than clinician‐administered diagnostic assessment. While incorporating patients' perspectives is important for understanding lived experience, this approach may reduce diagnostic reliability and limits comparability with studies employing standardised clinical diagnoses. Furthermore, the use of retrospective, self‐reported data may have entailed recall bias. On the other hand, with the passage of time, the participants could have formed a more reflective and balanced view of their treatment.

Researchers' positions inevitably influence the research process (Berger 2015). In this study, the absence of personal experience with EDs among the authors may have shaped both the interviews and the analysis. The sample was predominantly female, not reflecting the actual gender distribution of EDs (van Eeden et al. 2021). Moreover, aside from one participant treated abroad, all were White individuals of Finnish origin, limiting the generalisability of the results to other populations.

Because participation was voluntary rather than randomly assigned, self‐selection bias may have influenced the sample. Participants aware of the study's focus on treatment experiences may have been motivated by specific agendas, such as expressing dissatisfaction with care. Those with repeated unsuccessful treatments and ambivalence towards treatment might have been more inclined to attribute blame externally, although individuals who self‐identified as recovered reported similar experiences to those with ongoing illness. The reliance on a single interview also increases the potential for bias, though participants were invited to provide additional input afterwards. To enhance credibility, we employed strategies such as thorough record‐keeping, researcher reflexivity and a team‐based design. Finally, as the data reflect participants' subjective experiences, they may not fully represent the healthcare system; nonetheless, these perspectives are vital for improving treatment responsiveness and effectiveness.

## 6 Future Directions

The present findings provide valuable insight for the development and refinement of care services, while also identifying key areas for future research. There is a pressing need to reorient the current treatment paradigm to place greater emphasis on eating behaviour rather than body weight as the primary focus. Additionally, clinicians should remain acutely aware of the profound impact their attitudes and behaviours can have on patients' experiences and recovery trajectories. The healthcare system faces the critical task of developing individualised treatment pathways that ensure continuity of care and promote sustainable, long‐term outcomes. Future research should explore strategies for balancing clinical structure and control with sufficient flexibility, particularly in the context of inpatient and involuntary treatment, to reduce ambivalence and strengthen engagement.

## Funding

The study was supported by the Finnish Cultural Foundation (Grant No. 65221659) and the Signe and Ane Gyllenberg Foundation (Grant No. 5671) as personal working grants for this study.

## Ethics Statement

Ethical approval was obtained from the Ethics Committee of the Hospital District of Northern Savo (2191/2022), and the study was conducted in accordance with the principles of the Declaration of Helsinki.

## Consent

All participants gave their written, informed consents.

## Conflicts of Interest

The authors declare no conflicts of interest.

## Supporting information


Supporting Information S1

Supporting Information S2

## Data Availability

Data available on request due to privacy restrictions: The data that support the findings of this study are available on request from the corresponding author. The data are not publicly available due to privacy and ethical restrictions.
