# Supplementary material for: Severe and Enduring Eating Disorders: A Qualitative Study of Patients' Experiences and Perceived Treatment Needs
Source: Eur Eat Disord Rev. 2026 Jun 1;34(6):1454–64. doi: 10.1002/erv.70132 (PMC13656760; doi:10.1002/erv.70132)
Supplement: Supplementary file 1 [file ERV-34-1454-s001.pdf]

The interview guide

INTERVIEW WITH A PERSON WITH A SEVERE AND ENDURING EATING DISORDER

Date ________________________________

Time and place ________________________________

Participant ________________________________

Other ________________________________

Introductions: Researcher introduces herself, reviews interview process and how long the interview will approximately last.
Study purpose and applications: Researcher goes over the study's purpose and uses of the findings.
Consent form and approval: Informed consent form signed by the participant prior to the interview and assurance of privacy, confidentiality and anonymity will be reviewed. Related questions will be answered and mentioned once more that the interview will be recorded.
Treatment of data: Researcher indicates how data will be managed, secured, and destroyed.
Other concerns: Other issues are discussed prior to the beginning of the actual interview.

Opening words and general format for questions: Researcher explains that the purpose is to hear about the participant's personal experience with the topic and how they describe that experience by sharing their personal stories, insights, and interpretation of those experiences.

Interview questions

1. theme: Current state
    a. How are you doing now?
    b. Does your eating disorder impact your current life and if so, how?
    c. What is your life situation otherwise (Questionnaire Q1-9)
2. theme: Somatic health
    a. Do you know your height and weight? Do you follow your weight?
    b. Do you want to share your weight?
    c. How do you experience your physical health now?
    d. Has your weight changed during your eating disorder and if so, how? (Possible weight changes to be described by drawing a graph together with the subject)
3. theme: Treatment experiences
    a. What kind of treatment have you had for your eating disorder? (Questionnaire Q13-14)
    b. How would you describe the nutritional treatment that you have received? (Questionnaire Q15)

c. How long periods have you been outside of eating disorder treatment after first receiving treatment? (Questionnaire Q16)
d. What kind of experiences do you have concerning eating disorder treatment? Possible also, how have you experienced involuntary treatment? (Questionnaire Q17)
e. Which factors in treatment have helped you to get better?
f. Are there factors in treatment that have hindered recovery?
g. How does your other mental health disorders and/or neuropsychiatric disorders impact treatment? (Questionnaire Q18-20)
h. How does your somatic health conditions impact your treatment? (Questionnaire Q21-22)
i. How have you experienced therapists' attitudes and willingness to work for your recovery in different stages of treatment?
j. Have treatment targets changed during different treatments? Are you or the staff responsible for these changes?

4. theme: Needs for treatment and expectations for the future
    a. What do you think constitutes a good life?
    b. How could treatment improve your quality of life?
    c. What kind of support would you want and how much?
    d. What kind of wishes and needs do you have concerning your treatment?
    e. What kind of life goals do you have in general?

Final question: To get your final thoughts, is there anything else you would like to share with me regarding the topics we have discussed today?

Summary and the completion of the interview: Thanking the participant and the explanation of the possible follow-up by phone.
