# Supplementary material for: Severe and Enduring Eating Disorders: A Qualitative Study of Patients' Experiences and Perceived Treatment Needs
Source: Eur Eat Disord Rev. 2026 Jun 1;34(6):1454–64. doi: 10.1002/erv.70132 (PMC13656760; doi:10.1002/erv.70132)
Supplement: Supplementary file 2 [file ERV-34-1454-s002.pdf]

The questionnaire

I Background information

1. Gender
    a. female
    b. male
    c. other
    d. I don’t want to answer
2. Year of birth ___________
3. Children
    a. I don’t have children
    b. I have one or more children
    c. I am pregnant
4. Current occupation (over the past 12 months)
    a. employed
    b. unemployed jobseeker
    c. student
    d. on sick leave
    e. on parental leave
    f. retired
    g. other ___________
5. Frequency of work/studies (over the past 12 months)
    a. working 50 % or more
    b. working less than 50 %
    c. studying 50 % or more
    d. studying less than 50 %
    e. working and studying 50 % or more
    f. working and studying less than 50 %
    g. not working or studying

6. Main source of income (over the past 12 months)
    a. salary from employment or entrepreneurship
    b. unemployment benefit
    c. study grant
    d. sickness allowance
    e. parental allowance
    f. pension
    g. social assistance
    h. financial support from a close friend or relative
    i. own assets
    j. no source of income
7. The highest educational level
    a. comprehensive school
    b. general or vocational upper secondary school
    c. university or university of applied sciences
    d. none
8. Living situation (over the past 12 months)
    a. living alone
    b. living with a partner
    c. living with a child or children
    d. living with a partner and a child or children
    e. living with a parent or parents
    f. living with a parent or parents and an own child or children
    g. living with another adult with or without their children
    h. living with another adult and an own child or children
9. Marital status
    a. married
    b. living with a partner
    c. divorced
    d. widow
    e. in a relationship
    f. single

II Treatment history

10. The difficulties concerning weight, body or eating first started affecting my everyday life when I was __________ years old
11. I first received treatment for my eating disorder when I was __________ years old
12. I have been in treatment for my eating disorder altogether __________ years
13. I have received following treatments for my eating disorder
    a. visits at school or student health care or at the local health station
    b. health monitoring at school or student health care or at the local health station
    c. nutritional therapy
    d. physical therapy
    e. functional therapy
    f. psychotherapy
    g. drug therapy
    h. brain stimulation therapy
    i. other __________
14. I have been in the following treatment for my eating disorder
    a. outpatient treatment
    b. day-hospital treatment
    c. inpatient treatment
15. I have received the following nutritional rehabilitation for my eating disorder
    a. I have met with a nutritionist/dietitian
    b. a meal plan has been made for me
    c. I have been prescribed oral nutritional supplements
    d. I have received enteral tube feeding
    e. I have received parenteral nutrition
16. After first receiving treatment for my eating disorder there have been periods when I have not been in treatment for my eating disorder
    a. yes
    b. no
17. I have been ordered to treatment against my will
    a. yes
    b. no
18. I have other diagnosed mental health problems that I have received treatment for
    a. yes

b. no

19. If your answer in the previous question (18.) was “a. yes “, which diagnosed mental health problems:

____

20. I have been diagnosed with the following neuropsychiatric disorders:

a. none
b. autism spectrum disorder
c. dysphasia or a learning disability
d. Tourette syndrome
e. ADHD or ADD
f. other __________

21. I have the following somatic health conditions:

a. dental problems
b. gastrointestinal problems
c. food allergies
d. type 1 diabetes
e. type 2 diabetes
f. heart conditions
g. endocrinological disorder (e.g., hypothyroidism)
h. recurrent pain
i. bariatric surgery
j. osteopenia or osteoporosis
k. physical disability
l. leukopenia
m. none
n. other __________

22. If you have any of the somatic health problems or conditions listed in the previous question (21.), have they affected your eating disorder treatment?

a. yes
b. no
